# What the Cardiologist Needs to Consider in the Management of Oncologic Patients with STEMI-Like Syndrome: A Case Report and Literature Review

**DOI:** 10.3390/ph14060563

**Published:** 2021-06-12

**Authors:** Aneta Aleksova, Giulia Gagno, Alessandro Pierri, Carla Todaro, Alessandra Lucia Fluca, Valentina Orlando, Alessandra Guglielmi, Antonio Paolo Beltrami, Gianfranco Sinagra

**Affiliations:** 1 Cardiothoracovascular Department, Azienda Sanitaria Universitaria Giuliano Isontina (ASUGI), 34149 Trieste, Italy; gagnogiulia@gmail.com (G.G.); alessandro.pierri92@libero.it (A.P.); alessandralucia.fluca@units.it (A.L.F.); gianfranco.sinagra@asugi.sanita.fvg.it (G.S.); 2 Department of Medical Surgical and Health Science, University of Trieste, 34149 Trieste, Italy; carla.todaro@asugi.sanita.fvg.it; 3 Department of Perioperative Medicine, Intensive Care and Emergency, Azienda Sanitaria Universitaria Giuliano Isontina (ASUGI), 34149 Trieste, Italy; 4 Oncology Department, Azienda Sanitaria Universitaria Giuliano Isontina (ASUGI), 34149 Trieste, Italy; valentina.orlando@asugi.sanita.fvg.it (V.O.); alessandra.guglielmi@asugi.sanita.fvg.it (A.G.); 5 Department of Medicine (DAME), University of Udine, 33100 Udine, Italy; antonio.beltrami@uniud.it

**Keywords:** 5-Fluorouracil, acute myocardial infarction, acute coronary syndrome, ST-segment elevation myocardial infarction—STEMI, coronary spasm, myocardial enzymes, troponin I, chest pain, cardiotoxicity

## Abstract

In pre-hospital care, an accurate and quick diagnosis of ST-segment elevation myocardial infarction (STEMI) is imperative to promptly kick-off the STEMI network with a direct transfer to the cardiac catheterization laboratory (cath lab) in order to reduce myocardial infarction size and mortality. Aa atherosclerotic plaque rupture is the main mechanism responsible for STEMI. However, in a small percentage of patients, emergency coronarography does not reveal any significant coronary stenosis. The fluoropyrimidine agents such as 5-Fluorouracil (5-FU) and capecitabine, widely used to treat gastrointestinal, breast, head and neck cancers, either as a single agent or in combination with other chemotherapies, can cause potentially lethal cardiac side effects. Here, we present the case of a patient with 5-FU cardiotoxicity resulting in an acute coronary syndrome (ACS) with recurrent episodes of chest pain and ST-segment elevation.. Our case report highlights the importance of widening the knowledge among cardiologists of the side effects of chemotherapeutic drugs, especially considering the rising number of cancer patients around the world and that fluoropyrimidines are the main treatment for many types of cancer, both in adjuvant and advanced settings.

## 1. Introduction

Fluoropyrimidine agents such as 5-FU and capecitabine are extensively used in the palliative and adjuvant treatment of numerous solid tumors. In addition to systemic applications, 5-FU is often used topically for the treatment of various skin conditions.

A large body of research indicates that the toxicity induced by these two fluoropyrimidines is probably dose-dependent and mainly due to the efficiency of the dihydropyrimidine dehydrogenase (DPYD), which is the enzyme that catalyze 5-FU degradation [1,2]. Usually fluoropyrimidine toxicity is manageable, consisting of mild nausea, vomiting, diarrhea and myelosuppression [1]. A less frequent and underestimated complication, is cardiotoxicity [3]. Polymorphisms of DPYD and pre-existing heart disease are the main factors influencing the incidence of cardiotoxicity [1,4]. Cardiotoxicity could manifest as angina pectoris, myocardial infarction, atrial and ventricular arrhythmias, hypertension, hypotension, dilated cardiomyopathy and heart failure.

Interestingly, 5-FU-induced coronary vasospasm can result in all forms of ACS, including transient and persistent ST-segment elevation. After the suspension of the 5-FU, its re-introduction should be specifically made on a case-by-case basis for each patient. In particular, it can be proposed in association with CCBs prophylaxis and telemetry monitoring for younger patients in a context of curative chemotherapy.

Here, we present a case of an 80-year-old man with an intermittent ST-segment elevation ACS after treatment with 5-FU.

## 2. Case Presentation

**Figure 1:**
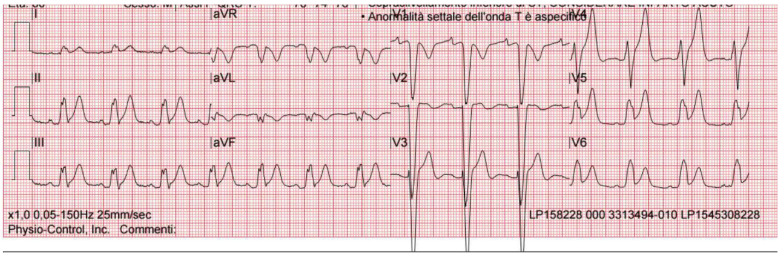
First EKG recorded by 118 (markedly agitated patient), sinus rhythm, left bundle branch block, inferolateral subepicardial lesion.

An 80-year-old man, non-smoker, with a history of grade 1 hypertension and a known left bundle branch block (LBBB) was directly transferred to the catheterization laboratory (cath lab), bypassing the Emergency Department (ED) for urgent coronary angiography, due to severe, prolonged chest pain irradiating to the left arm and associated with the presence of ST-elevation over inferior and lateral leads, in addition to the known LBBB at pre-hospital electrocardiogram (EKG) (Figure 1). The patient had no other cardiovascular risk factors and was not taking any cardiovascular medication.

The patient reported that a few hours before the admission he had experienced a transient episode of chest pain which he had attributed to muscle strain.

Upon arrival, he appeared uncomfortable, but no signs of pulmonary congestion were detected, with the rest of the cardiovascular examination appearing normal. Forty-eight hours before admission the patient had begun first line chemotherapy with a modified FOLFOX regimen (consisting of a combination of 5-FU, Leucovorin and Oxaliplatin administered as in-hospital bolus, plus 46 h of continuous infusion through Port-a-Cath, every two-weeks) to treat a stage IV colorectal cancer with hepatic metastasis. The patient’s vitals were stable: blood pressure at 125/85 mmHg, heart rate of 65 beats per minute, respiratory rate of 16 breaths per minute, oxygen saturation at 99% while breathing in ambient air. A dual antiplatelet therapy with a loading dose of acetylsalicylic acid (250 mg), ticagrelor (180 mg) along with an intravenous (iv) bolus of heparin sodium 4000 U were also administered during the ambulance transfer.

**Figure 2:**
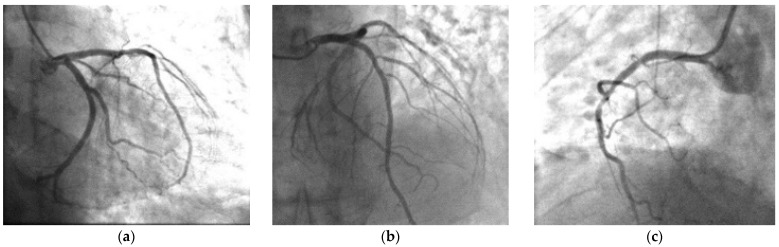
Cardiac catheterization. Right anterior oblique, (**a**) caudal and (**b**) cranial of left coronary anatomy (stenosis 40% on the first diagonal). Left anterior oblique, (**c**) cranial of right coronary anatomy (stenosis 50% on the proximal right coronary).

During the preparation for the procedure in the cath lab, the patient’s pain subdued. The emergency coronary angiogram did not reveal any hemodynamically significant stenosis of the coronary tree (Figure 2). There was a focal 40% stenosis of the first diagonal and a 40% focal stenosis of proximal circumflex artery, which were considered not hemodynamically significant.

**Figure 3:**
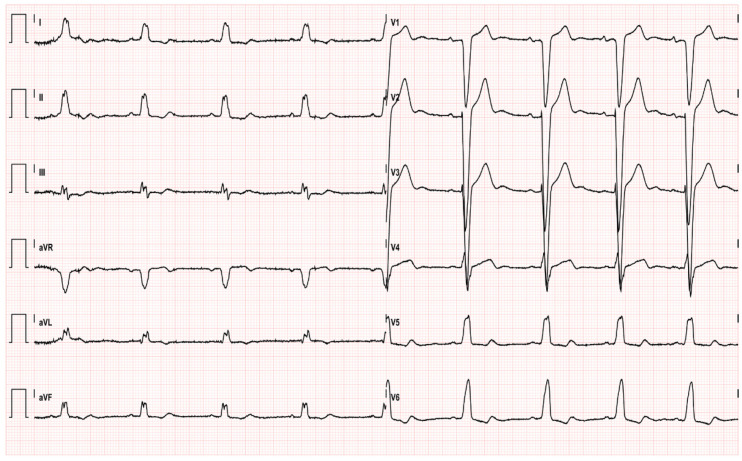
EKG after emergent coronarography; sinus rhythm, heart rate 55 bpm, LBBB, ST normalization.

EKG performed afterwards showed normal sinus rhythm with LBBB, without ST-segment abnormalities that could indicate ischemia (Figure 3).

The patient was transferred to the Intensive Cardiac Care Unit (ICCU) for further care and monitoring. Transthoracic echocardiography showed overall normal biventricular systolic function.

**Figure 4:**
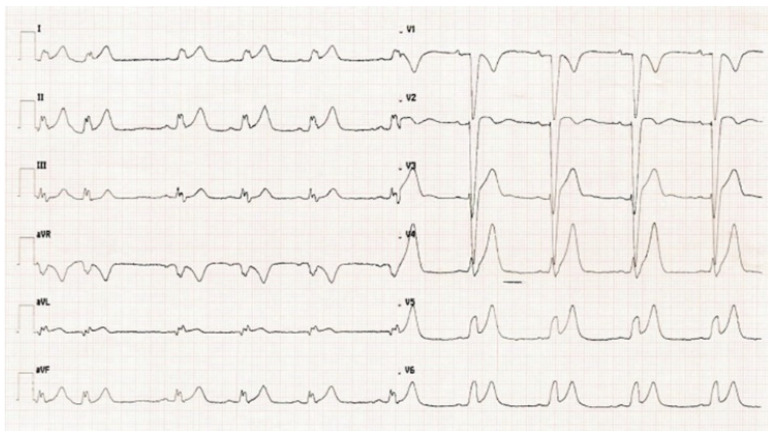
EKG during second chest pain episode.

Unfortunately, the infusion of 5-FU was not suspended and approximately one hour after his admission to ICCU, the cardiac intensivist had to be called in because the patient was experiencing another episode of intense chest pain. On 12 lead EKG (Figure 4) as well as on EKG-telemonitoring, sinus rhythm, LBBB and inferolateral subepicardial ischemia were detected. A transthoracic echocardiogram showed a mild reduction in left ventricular function with an ejection fraction (EF) of 43% and hypokinesis of inferior, septal and apical walls.

**Figure 5:**
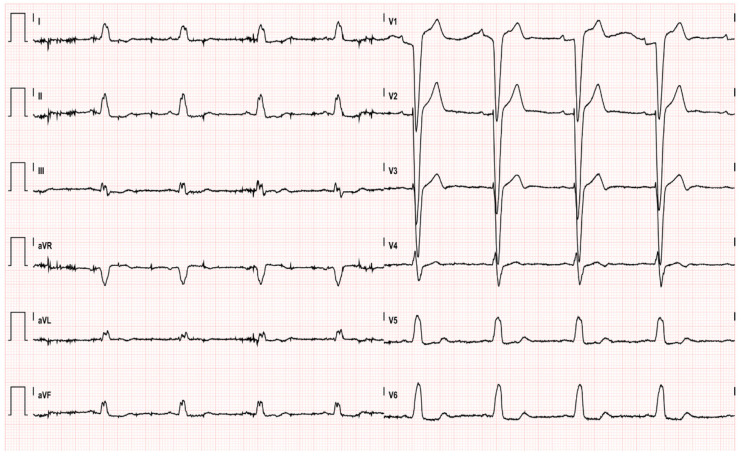
EKG after diltiazem administration.

As soon as it was understood that the arising coronary spasms were 5-FU-induced, chemotherapy was immediately suspended. Symptoms and EKG changes were not responsive to the initial treatment with iv bolus nitrates (5 mg) and continuous nitrate infusion. Consequently, despite the relatively low heart rate (65 beats per minute), iv bolus diltiazem (15 mg) was promptly administered with a subsequent improvement in symptoms and a gradual resolution of ST elevation (Figure 5).

Once the patient’s symptoms stabilized following the iv therapy, diltiazem was orally administered at a lower dose of 30 mg, three times per day due to the patient’s relatively low heart rate. The dosage of diltiazem was therefore up-titrated to the maximum tolerated dose of 60 mg every 8 h, with a gradual reduction and complete disappearance of angina attacks (during the first 2 days in ICCU he experienced three episodes of chest pain associated with ST segment elevation in inferolateral leads). High sensitivity troponin did not increase significantly over time (peak troponin level 28 ng/L) and other standard laboratory tests showed results within normal thresholds (hemoglobin 12.8 g/dl, platelet count 237,000/mmc, glycemia 118 mg/dl, serum creatinine 0.91 mg/dl, serum sodium 139 mEq/l, serum potassium 3.86 mEq/l). No arrhythmias were recorded during ICCU stay.

He was discharged after six days. Taking into consideration the patient’s age, palliative chemotherapy aim and the high risk of relapsing myocardial ischemia, re-challenge with 5-FU was not performed and an alternative chemotherapy regimen was started instead. At six months follow-up the patient reported no chest pain relapses nor echocardiographic changes were detected, therefore no other exams were planned.

## 3. Discussion

### 3.1. Indications for 5-FU

Fluoropyrimidine agents, such as 5-FU and Capecitabine, are widely used in the palliative and adjuvant treatment of several solid tumors. Labeled indications include gastrointestinal, breast, head and neck cancers, either as a single agent or in combination with other chemotherapies. Beyond the Food and Drug Administration (FDA)-approved indications, 5-FU is frequently employed off-label in hepatic and genitourinary cancers.

In addition to systemic applications, 5-FU is often used topically for the treatment of various skin conditions [3]. Approved by the FDA for the treatment of actinic or solar keratoses and superficial basal cell carcinoma, topical 5-FU has also demonstrated efficacy in cutaneous melanoma metastases, keratoacanthoma and conventional treatment-resistant vitiligo.

### 3.2. 5-FU Toxicity and Risk Stratification

Usually, fluoropyrimidine toxicity is manageable, consisting of mild nausea, vomiting, diarrhea and myelosuppression, except for DPYD polymorphisms [5]. The DPYD gene encodes for an enzyme that catalyzes the rate-limiting step in fluorouracil catabolism, so that some DPYD genetic variants can lead to a decreased enzymatic activity, with implications in terms of toxicity. Therefore, Iachetta et al. have suggested a systematic DPYD genotyping before the onset of chemotherapy [6]. The stratification of patients on the basis of the DPYD genotype may prevent such adverse events, through the administration of a reduced dose of the chemo-drug in patients who are carriers of pathogenic variants.

A routine pre-treatment screening of deleterious DPYD polymorphisms is rarely adopted in clinical practice, although the main current guidelines of the Clinical Pharmacogenetics Implementation Consortium have identified the four clearly pathogenic variants associated with 5-FU toxicity [7]. These DPYD polymorphisms (c.1905 + 1G > A, rs3918290; c.2846A > T, rs67376798; c.1679T > G; rs55886062 and c.1236G > A, rs56038477), which are the most commonly observed in the Indo-European population, can be detected using LAMP Human DPD deficiency kit (LaCAR MDx Technologies). Moreover, genotypic tests for DPYD variants before 5-FU administration may be cost-effective: in a large-scale analysis, Deenen et al. show that upfront genotyping of DPYD*2A variant—also known as rs3918290—is feasible and cost-saving [8]. Indeed, DPYD*2A is the most relevant polymorphism responsible for DPYD deficiency and its prevalence is 1% to 2% in the Western population [9], so that screening costs are substantially offset by the reduction in toxicity-related patient charges.

A less frequent and underestimated, but potentially lethal side effect, is cardiotoxicity [10]. For example, in this case report, the diagnosis of 5-FU induced vasospasm was at first overlooked by the cardiologist on call and the initial management included an emergency coronary angiography. To date no clear risk factors are known to predict and prevent cardiotoxicity in “healthy” patients and the reported incidence of this side effect can range from 4% to 30.6%, according to reports from oncologists and cardiologists, respectively [11,12]. On the other side, pre-existing heart disease and an impaired renal function seem to be the most important risk factors associated with 5-FU cardiotoxicity [4], without forgetting the role of genetic susceptibility.

Another controversial topic about 5-FU toxicity concerns combination treatments: could 5-FU toxicity be enhanced by other chemo-drugs or radiotherapeutic treatment? While Labianca et al. found that the concomitant treatment with other chemo-drugs, such as vincristine, cyclophosphamide and methotrexate did not alter the incidence of 5-FU cardiotoxicity [13], in another study the association among capecitabine, oxaliplatin and bevacizumab led to higher risks of cardiotoxicity if compared with capecitabine monotherapy [14]. Oxaliplatin, used in conjunction with 5-FU in our patient, is not known to cause such side effects on its own, but there is the possibility it might have enhanced 5-FU cardiotoxicity. Regarding radiation therapy, the previous myocardial irradiation was proposed as an additional risk factor of 5-FU cardiotoxicity [15].

Lastly, the dose dependence of 5-FU-related cardiotoxicity is an area of ongoing debate [2], even though circulating FU levels seemed not to correlate with cardiovascular side effects in a study by Thyss et al. [16]. On the contrary, a relationship between the dosage of 5-FU and other FU-associated toxicities has been documented, for example, where the severity of intestinal injury is associated with the dosage of 5-FU in a murine model [17].

### 3.3. 5-FU Cardiotoxicity from a Clinical Point of View

Cardiotoxicity could manifest itself as angina pectoris, atrial and ventricular arrhythmias, hypertension, hypotension, dilated cardiomyopathy and chronic heart failure. Acute heart failure remains a relatively rare occurrence [18], even if occasional cases of cardiogenic shock are reported in literature [19]. However, left ventricular dysfunction, including echocardiographic views that copy Takutsubo syndrome, may often be observed [20,21]. Some recent reports even describe cases of severely reduced ejection fraction recognized accidentally during the diagnostic workup, in which the patient did not experience any symptoms or signs [22]. These reports emphasize the importance of close monitoring during the first phase following 5-FU therapy. In fact, an acute left ventricular systolic dysfunction, although asymptomatic and reversible, increases the risk for complications like thrombus formation and fatal arrhythmias.

The monitoring should include at least an EKG and the assessment of serum biomarkers of myocardial injury. In particular, the evaluation of N-terminal pro-brain natriuretic peptide (NT-proBNP) may facilitate safer applications of this cardiotoxic drug [23]. NT-proBNP elevation, generally associated with hemodynamical stress caused by contractility abnormalities, could also be the result of a direct toxic effect on myocardial cells. However, a normal level of NT-proBNP, as for the other common biomarkers of cardiac injury (troponin and creatine phosphokinase), does not rule out cardiotoxicity, showing a low negative predictive value [24].

Moreover, EKG changes and arrhythmias can also occur in asymptomatic patients [25,26,27]. Both silent repolarization and rhythm disorders pose an important management challenge for the cardiologist, due to their unclear clinical significance.

Fortunately, the cardiac adverse effects of fluoropyrimidines can be usually reversed through the discontinuation of the treatment [28,29,30,31].

### 3.4. Underlying Pathogenic Mechanisms

According to the available literature, the most common manifestation of 5-FU-induced cardiotoxicity is myocardial ischemia, usually occurring at rest and characterized by ST segment elevation following coronary vasospasms. Well-documented reported cases of acute coronary syndrome (ACS) due to spasm (proved or likely) in the setting of fluoropyrimidine use are summarized in Table 1.

The relevance of this pathogenic mechanism of acute coronary syndrome in several patients receiving 5-FU is supported by coronary angiography (not infrequently evidence of normal coronaries [32,33,34,35] and occasionally a direct visualization of coronary spasms) [36,37] and clinical course (a sudden onset and rapid recovery from symptoms, transitory ischemic changes in EKG, responsiveness to anti-spasmogenic therapy).

As in the case described above, the symptoms and instrumental abnormalities are solved through the discontinuation of fluoropyrimidine drug treatment together with diltiazem administration, even if they transiently reappear in the following days in accordance with the disposal time of 5-FU [38,39]. Therefore, patients need to continue intensive monitoring for several days after withdrawal. To date, it is known that 5-FU plasma’s half-life is relatively short (around 4 h) since it is catabolized by liver enzymes, especially DPYD. Nevertheless, it does not avoid tissue accumulation, leading to the formation of active 5-FU metabolites, which explains the long-lasting cytotoxicity [40]. The mechanisms of 5-FU induced cardiotoxicity are not completely understood and coronary vasospasm seems to be the most well documented manifestation [41,42].

**Figure 6:**
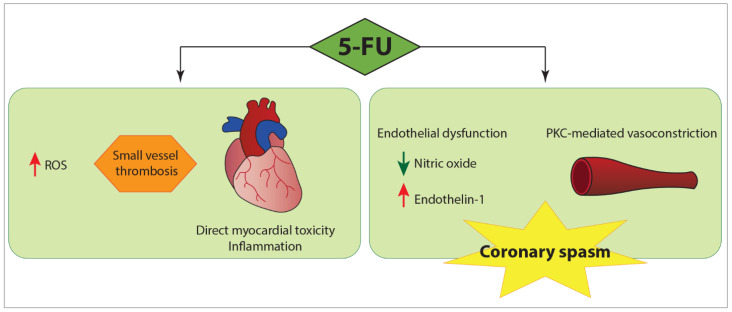
Possible mechanisms of 5-FU cardiotoxicity. Despite coronary vasospasm being the most well-established manifestation of 5-FU cardiotoxicity, a multifactorial mechanism has been postulated, involving both ischemia secondary to coronary vasospasm and direct myocardial damage. 5-FU, 5-Fluorouracil; PKC, protein kinase C; ROS, reactive oxygen species.

The mechanism behind spasms is complex and still unclear, but it is likely linked to an endothelial dysfunction with imbalances between local vasoconstrictors and vasodilators (Figure 6). For example, increased plasma levels of endothelin-1 were detected in patients receiving 5-FU [43,44], whereas the deficiency in nitric oxide amplified the hyperreactivity of cardiac arteries [45]. In addition, an endothelium-independent mechanism has been hypothesized, demonstrating in vitro protein kinase C-mediated vasoconstriction of vascular smooth muscles [42]. However, in some patients abnormalities of the left ventricular wall motion have been reported in areas not corresponding to coronary vessel distribution, suggesting a multifactorial cardiotoxicity mechanism: the occurrence of ischemia, secondary to coronary vasospasms, is only a component of the pathophysiological spectrum, which also includes the thrombosis of small vessels, direct toxicity on the myocardium and inflammation that could lead to myocarditis [46]. For example, Calik et al. described an original case of acute toxic myopericarditis occurring after the first bolus dose of 5-FU [47]. Their patient, admitted to the ICCU with clinical features similar to ours, presented however inflammatory biomarkers elevation and echocardiography revealed global myocardial hypokinesia of the left ventricle. Therefore, she was treated with ibuprofen, metoprolol and ramipril and discharged without any problems, showing a complete normalization of systolic function after 2 months.

The evidence that some patients develop global electrocardiographic changes and generalized left ventricular hypokinesia suggests the possibility of a 5-FU induced global myocardial abnormality.

Sometimes more pathogenic mechanisms can coexist: Dalzell and Samuel describe a case of 5-FU cardiotoxicity where symptoms and instrumental findings cannot be explained either by coronary spasm or by myocarditis alone [48].

A 5-FU induced increase in reactive oxygen species (ROS) has also been documented. Focaccetti et al. examined the effects of 5-FU on cell cultures of human cardiomyocytes and endothelial cells, observing that ROS elevation characterized the endothelial response [49].

### 3.5. Management of 5-FU Acute Coronary Syndrome with ST Segment Elevation

**Figure 7:**
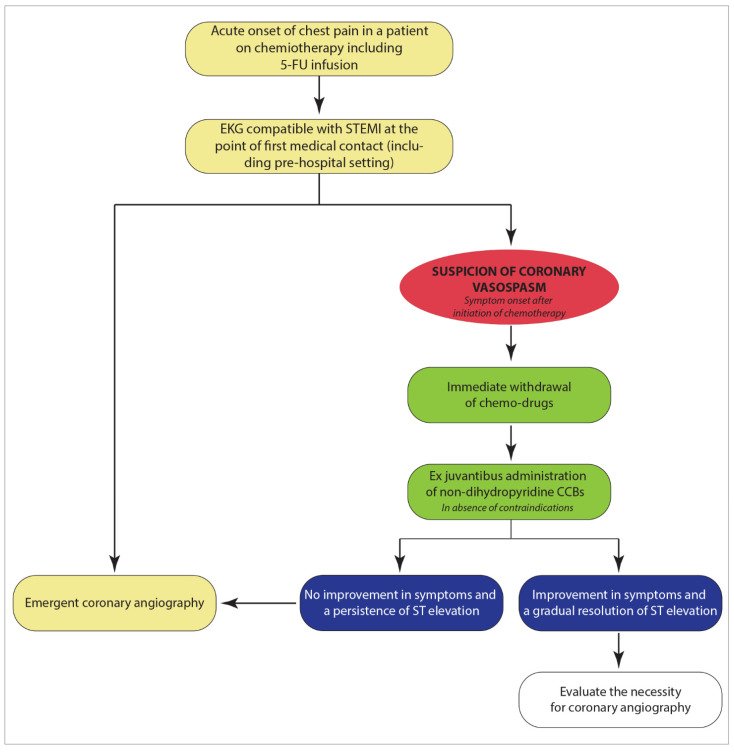
Diagnostic−therapeutic pathway of STEMI in patients undergoing 5-FU infusion.

Based on these pathophysiological considerations, calcium channel antagonists appear very useful in the context of coronary spasms, in particular non-dihydropyridine calcium channel blockers (CCBs). In the hypothesis of coronary vasospasm with patients receiving treatment with fluoropyrimidine agents, the administration of CCBs and nitrates should be considered as a first-line ex juvantibus treatment in a pre-hospital setting, as well as in spoke hospitals, before transferring the patients to the hub center for emergency coronary imaging (Figure 7). Clearly, as demonstrated by our case, the tendency to bradycardia could represent a limit to both the introduction and up-titration of CCBs. However, in the case of excessive bradycardia with diltiazem or verapamil, a shorter-acting substance such as the dihydropyridine nifedipine should be tested instead. On the other hand, beta-blockers should be avoided in these patients due to their spasmogenic power.

In any case, in the context of ST-segment elevation myocardial infarction (STEMI), cardiac catheterization remains an overriding exam: the absence of obstructive atheromatous coronary artery disease will strengthen the association among clinical features and chemotherapy. On the other side, a wrong attribution of the clinical picture to chemotherapy can lead incorrectly to a discontinuation of the drug, compromising the cancer treatment.

Even though most cases of cardiac side effects of 5-FU improve spontaneously, recurrence is possible when the drug is administered again to the same patient. From this point of view, two important issues should be discussed: prophylactic treatment and re-challenge with 5-FU.

Coronary spasm being the most common physiopathological mechanism behind 5-FU cardiotoxicity, the possible usefulness of a spasm provocation test with ergonovine (ER) or acethilcoline (Ach) could be imagined. However, these tests are not strongly recommended in current ESC guidelines and should be limited to selected patients [50]. In our case, since symptoms and EKG alterations had resolved after 5-FU withdrawal and diltiazem treatment, an “ex adjuvantibus” diagnosis was made according to the clinical context, without the need for provocative tests. Furthermore, with the benefit of hindsight, the spasm provocation test would have probably exposed the patient to a significant additional risk in the acute phase, since ER and Ach could cause life-threatening side effects [51], potentially exacerbated in combination with 5-FU. Finally, as stated above, coronary spasm is not the only mechanism of 5-FU cardiotoxicity. Therefore, we can speculate a negative coronary spasm test to be not enough to rule out 5-FU cardiotoxicity. Accordingly, in view of all these considerations, a provocative test for coronary artery spasm was not deemed appropriate for this patient.

### 3.6. Profilaxis

The prophylactic role of calcium channels has been studied but its exact role is still debated. In a study from Eskilsson and Albertsson, the prophylactic verapamil was used on 58 patients treated with 5-FU for esophageal, head and neck cancers [52]. In this study Verapamil did not reduce the insurgence of myocardial ischemia during chemotherapy treatment. However, if compared to the historical control, it seemed to be useful in preventing arrhythmias, which have been associated with a more malignant course.

In another study the administration of nitrates and/or calcium channel blockers failed to prevent recurrence of 5-FU cardiotoxicity [53]. These results do not rule out the possibility of coronary artery spasm but suggest a heterogeneous response to a vasodilator among patients. After all, as stated above, several mechanisms other than spasm may underpin 5FU-cardiotoxicity, thus explaining the inefficacy of a vasodilator.

In a few cases, the positive effects of calcium channel blockers prophylactic treatment have been reported, but the dosage and right timing of the administration still have to be investigated [54]. Furthermore, some calcium channel blockers such as nifedipine may be dangerous, for example, enhancing the toxic effects of concomitant chemo-drugs [55].

### 3.7. Re-Challenge with 5-FU

Another controversial issue concerns the potential re-challenge with 5-FU: studies have shown that the drug’s re-introduction after 5-FU-induced ACS can be successful [55,56,57,58]. However, the choice of attempting a re-challenge should be specifically made on a case-by-case basis for each patient. In particular, it can be proposed in association with CCBs prophylaxis and telemetry monitoring for younger patients in a context of curative chemotherapy.

For what concerns our patient, the risk/benefit ratio did not justify the adoption of this therapeutic option, therefore alternative treatments were chosen [59].

## 4. Take Home Message

Knowledge advancements and the increasing complexity of care specialization have led to many points of contact between different branches of medicine. In the context of a multifaceted healthcare system, a teamwork approach is crucial to improve patient safety and overall outcome. Our case report highlights the importance of broadening the understanding among cardiologists of chemotherapeutic drugs’ side effects. Thus, if life-threatening cardiac injury from chemotherapy is suspected, the treatment should be suspended as soon as possible. Furthermore, in these cases an ex juvantibus treatment with CCBs and nitrates should be considered before any emergency coronary angiography.

## 5. Conclusions

This is a well-documented case of cardiotoxicity induced by fluoropyrimidine upon first administration in a patient with few cardiovascular risk factors. We strongly believe that all patients who receive fluoropyrimidines should be notified about the possibility of this type of toxicity and should be thus prompted to report symptoms to an oncologist in order to avoid major complications. The knowledge of the potential cardiotoxic effects of chemotherapeutic drugs is fundamental for every cardiologist in order to raise the clinical suspicion of cardiotoxicity. Moreover, a detailed discussion with the oncologist must be encouraged in order to evaluate the risk/benefit ratio of the treatment and any alternative option.

## Figures and Tables

**Table 1:** Previously well-documented described cases of ACS following coronary spasm (proved or likely) associated with fluoropyrimidine use. Differences in echocardiographic features and troponin levels are probably attributable to differences in coronary spasm extension and duration, even though other concomitant pathophysiological causes, such as myocardial inflammatory injury and vascular endothelial dysfunction, cannot be ruled out.

	Luwaert et al. [36]	Camaro et al. [34]	Dalzell and Samuel [48]	Atar et al. Patient 1 [33]	Atar et al. Patient 2 [33]	Shah et al. [37]	Dechant et al. [18]
AGE/GENDER	70/M	61/M	54/M	40/F	63/M	28/M	51/M
PRIOR CARDIAC HYSTORY	None	Mild coronary artery disease	None	None	Unspecified coronary artery disease	None	None
CARDIAC RISK FACTORS	Smoke, arterial hypertension	Not reported	Not reported	Not reported	Not reported	None	Smoke, arterial hypertension, PAD, hyperlipidemia
CANCER	Squamous carcinoma of the palate	Metastatic colorectal cancer	Colon adenocarcinoma	Adenocarcinoma of the cecum	Adenocarcinoma of the duodenum	Metastatic colorectal cancer	Rectal cancer
ADDITIONAL CHEMOTHERAPY	Carboplatin	None	Oxaliplatin, leucovorin	Folinic acid	Folinic acid	None	Non reported
MODE OF ADIMINISTRATION	5-FU infusion	Oral capecitabine	5-FU infusion	5-FU infusion	5-FU infusion	Oral capecitabine	5-FU bolus + infusion
DOSE	1000 mg/m^2^/day	1500 mg/m^2^ twice daily	NR	425 mg/m^2^/day	425 mg/m^2^/day	1250 mg/m^2^ twice daily	400 mg/m^2^ iv bolus followed by 2400 mg/m^2^ iv infusion.
SYMPTOMS	Angina pectoris	Retrosternal chest pain	Typical chest pain	Chest pain	Chest pain	Cardiac arrest (ventricular fibrillation)	Typical chest pain
TIMING OF ONSET SYMPTOMS	Day 3	Day 1	20 h into the infusion	Day 3	Day 3	5 of a total of 6 cycles of chemotherapy	Day 2
EKG	ST-segment elevation in inferolateral leads	ST-segment elevation in inferolateral leads and peaked T-waves	Lateral ST elevation with reciprocal change alternating with intermittent left bundle branch block	ST-segment elevation in leads II, III, aVF, V5 and V6	ST-segment elevation in leads II, III, aVF, V5 and V6	Post-ROSC:ST segment elevation in the inferolateral leads	Significant ST elevations and prominent T waves in almost all leads (I–III, aVF and V2–V6)
TROPONIN	Not reported	Normal	Elevated	Normal	Normal	Elevated	Not reported
ECHO	Normal	Normal	EF 30%, global hypokinesis	Normal	Normal	Normal	EF 24%, global hypokinesis
CATH	Normal coronaries, ergonovine test + for spasm	No changes in comparison with the previous angiography	Normal coronaries	Normal coronaries, cold pressor test + for spasm	Severe multivessel coronary artery disease, cold pressor test	Normal coronaries	Generally reduced coronary flow. after 2 days normal coronaries.
INTERVENTION	Diltiazem and nitrate	Nifedipine	Ramipril, Metoprolol	Diltiazem	Diltiazem and Nitrate	Verapamil, defibrillator	ACE inhibitor, Verapamil, diuretics

## Data Availability

Data is contained within the article.

## Abbreviations

5-FU	5-Fluorouracil
Ach	Acethilcoline
ACS	Acute coronary syndrome
Cath lab	Catheterization laboratory
CCBs	Calcium channel blockers
DPYD	Dihydropyrimidine dehydrogenase
ED	Emergency Department
EF	Ejection fraction
EKG	Electrocardiogram
ER	Ergonovine
FDA	Food and Drug Administration
ICCU	Intensive Cardiac Care Unit
LBBB	Left bundle branch block
NT-proBNP	N-terminal pro-brain natriuretic peptide
ROS	Reactive oxygen species
STEMI:	ST-segment elevation myocardial infarction
